# Comparing the effectiveness of two cardiovascular prevention programmes for highly educated professionals in general practice: a randomised clinical trial

**DOI:** 10.1186/1471-2261-13-38

**Published:** 2013-06-01

**Authors:** Neree Claes, Nele Jacobs, Els Clays, Ward Schrooten, Ilse De Bourdeaudhuij

**Affiliations:** 1 Faculty of Medicine, Hasselt University, Agoralaan Building D, Diepenbeek, 3590, Belgium; 2 Antwerp Management School, prof Health Care Management, Sint-Jacobsmarkt 9-13, Antwerpen, 2000, Belgium; 3 Department of Public Health, Ghent University, De Pintelaan 185, block A, Ghent, 9000, Belgium; 4 Ziekenhuis Oost-Limburg, Schiepse Bos 6, Genk, 3600, Belgium; 5 Department of Movement and Sports Sciences, Ghent University, Watersportlaan 2, Ghent, 9000, Belgium

**Keywords:** Cardiovascular diseases, Prevention, General practice, Lifestyle programmes

## Abstract

**Background:**

Cardiovascular disease is a major cause of mortality and morbidity and its prevalence is set to increase. While the benefits of medical and lifestyle interventions are established, the effectiveness of interventions which seek to improve the way preventive care is delivered in general practice is less so. The aim was to study and to compare the effectiveness of 2 intervention programmes for reducing cardiovascular risk factors within general practice.

**Methods:**

A randomised controlled trial was conducted in Belgium between 2007-2010 with 314 highly educated and mainly healthy professionals allocated to a medical (MP) or a medical + lifestyle (MLP) programme. The MP consisted of medical assessments (screening and follow-up) and the MLP added a tailored lifestyle change programme (web-based and individual coaching) to the MP. Primary outcomes were total cholesterol, blood pressure, and body mass index (BMI). The secondary outcomes were smoking status, fitness-score, and total cardiovascular risk.

**Results:**

The mean age was 41 years, 95 (32%) participants were female, 7 had a personal cardiovascular event in their medical history and 3 had diabetes. There were no significant differences found between MP and MLP in primary or secondary outcomes. In both study conditions decreases of cholesterol, systolic blood pressure, and diastolic blood pressure were found. Unfavourable increases were found for BMI (p < .05). A significant decrease of the overall cardiovascular risk was reported (p < .001).

**Conclusions:**

Both interventions are effective in reducing cardiovascular risk. In our population the combined medical and lifestyle programme was not superior to the medical programme.

**Trial registration:**

ISRCTN23940498

## Background

Cardiovascular disease (CVD) is a major cause of mortality and morbidity in industrialized countries and is accountable for high societal costs [1]. Because of the severe disease burden, preventive actions are perceived as a major public health priority. These actions are important, knowing that the contribution of preventable risk factors is 70-76% for the total number of CVD [2].

**Figure 1:**
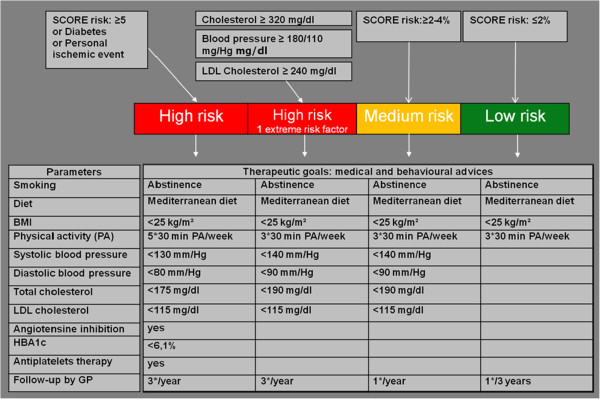
Guidelines for cardiovascular prevention.

In order to lower CVD, preventive programmes should focus both on medical (cholesterol, blood pressure, glycaemia) and lifestyle risk factors (smoking, low levels of physical activity, and unhealthy diet) [2]. Scientific evidence suggests that such programmes have favourable effects on cardiovascular (CV) risk factors [3-5]. Those programs and interventions are delivered by primary care providers (ea. trained nurses, dieticians, physiotherapist) with no/minimal intervention from the general practitioners. Literature review learned that the implementation of such programmes is rarely studied within general practice [6]. However disappointing follow-up of patient with CV risk factors in primary care - recently found in the Euroaspire survey - stressed out the urge to study the implementation of CV prevention programmes within general practice. Organisation of such preventive programmes has to be based on clinical guidelines and its implementation has to be done in line with Buckley’s criteria for general practice (recall of patients, systematically monitoring of risk factors and medication, patient and general practitioner education) (Figure 1) [7,8]. Those models and their implementation have not been studied on their effectiveness in general practice.

Our first aim was to study the effectiveness of two CV prevention programmes for reducing cardiovascular risk factors for highly educated professionals within general practice. Both programmes are based on the European guidelines for CV. Our second aim was to implement two prevention models feasible for general practice and based on Buckley’s implementation factors. The implementation of such programmes on a larger scale will be discussed in detail at the end of this paper.

## Methods

### Participants

**Figure 2:**
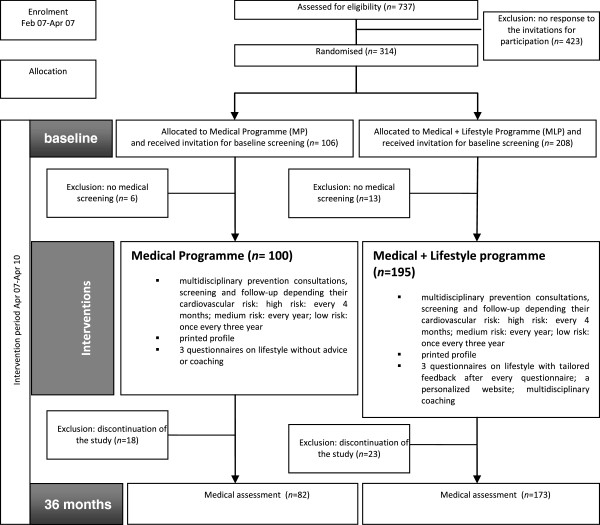
Study flow.

PreCardio was a randomised clinical trial with a follow-up of 3 years [9]. The inclusion criteria for participants were age between 25–75 years and being insured by the ‘De Onderlinge Ziekenkas’ and living in the province of Limburg. All of the insured were self-employed lawyers. This led to the selection of a highly educated and relatively healthy group of professionals participating in the study. This is a general sample of an active group of professionals. The selection of this specific group of professionals, namely self-employed lawyers, was because of to the good organisation of this professional group which is adequate to disseminate information on the study in an efficient way. All study participants signed an informed consent and had access to the internet. No other eligibility criteria were used. Study recruitment took place between February and April 2007. Potential participants (n = 737) were invited through an active recruitment strategy within the Bar (=professional organizations of lawyers): the president of the bar was informed on the study by the study-coordinator, secondly the study was presented at the board of directors, after their positive advice every director committed to convince 25 lawyers to participate. Supplementary, a letter from the study secretary with an invitation from the head of the lawyers was sent with the request to participate and to sign an informed consent. Three-hundred fourteen adults signed an informed consent to participate (42%). The study participants were randomized using a nonstratified randomization technique with a known probability. Each participant had a 67% chance to be allocated to the intervention group. The randomisation was performed by an independent person. The names of the participants were written on papers that were put in sealed envelopes. Next, the envelopes were randomly assigned by hand to two baskets for the intervention and the control group, respectively, with a ratio of 2/1. It was no blind randomisation. One hundred six participants were randomised to the MP and 208 to the MLP. However, 6 participants of the MP and 13 of the MLP chose not to start the study despite prior consent, resulting in 295 participants at the start of the study. The participant flow chart is shown in Figure 2. Of these participants 68% were male, the mean age was 41 years, the mean systolic blood pressure was 131 mmHg and the mean diastolic blood pressure was 84 mmHg. The mean cholesterol of the participants was 188 mg/dl, the mean body mass index (BMI) was 25 kg/m^2^ and 18% were smokers. Approval was obtained from the ethics committee of Hasselt University and the study was registered (ISRCTN23940498).

### Interventions

Participants in both prevention programmes (**MP + MLP**) were invited for a baseline **multidisciplinary prevention consultation** at Hasselt University, consisting of a medical screening by a general practitioner (GP) (anamnesis, blood pressure, cholesterolaemia, glycaemia), a dietician consult (weight, length and fat percentage), an assessment of physical fitness (step test) and a consult by a psychologist [9]. Patients were classified in the pre-contemplation, contemplation, preparation, action or maintenance stage [10]. After this screening all the participants received a **printed profile** with their individual risk factors and total cardiovascular risk [11]. This printed profile also contained advices to improve lifestyle factors based on all the results of the assessment. Participants with an aberrant medical risk factor (high blood pressure, hypercholesterelaemia or -glycaemia) and a high total risk were referred to **their GP** for immediate follow-up. Participants with a medium risk were advised to consult their GP every year, those with low risk, once every three year. The GPs of the participants were educated during a 4 hours training and had access to an online education tool for CVD prevention. An electronic risk calculator was developed and integrated in the electronic medical file of the GPs (n = 240) [12]. Supplementary a copy of the participants printed profile was sent to their GP. The multidisciplinary screening consultations were organised yearly at the University during the study. Participants of the **MLP** received additional interventions based on their personal scores for behavioural (physical activity, nutrition, smoking) variables [13-15]. Participants received access to a personalised website and one-on-one coaching. The **personalised website** included tailored advices based on the variables mentioned above. The **one-on-one coaching** targeted one or multiple behaviours based on the preferences of the individual. The coaching was delivered by a health psychologist in collaboration with a general practitioner, a physiotherapist, a cardiologist and assisted by students in sports or diet counselling. The coaching could be delivered by regular mail, e-mail, telephone, face-to-face (individual or group). Participants could freely determine the intensity and delivery mode of the MLP [16].

### Power calculation

A power calculation was performed to determine the number of study participants needed to detect a significant effect between the both programmes with a power of 80%. The sample calculation was based on the population standard deviation of the primary outcome measure. For our study this was the systolic blood pressure (SBP). Kelly et al. found a mean SBP of 125 mmHg (SD 14) [17]. The sample size calculation was performed with Nquery Advisor 4.0®. A two group t-test with a 0.05 two-sided significance level would have 80% power to detect the difference between the MP mean of 125 mmHg and the MLP mean of 120 mmHg. Assuming a common standard deviation of 14 mmHg, a total sample size of 282, 93 in the MP and 186 in the MLP would be sufficient.

### Outcome measures

The aim of this randomised trial was to examine the effectiveness of two cardiovascular prevention programmes in reducing cardiovascular risk factors within primary care with 3 years of intervention (April 2007-April 2010). The primary outcomes were systolic and diastolic blood pressure, total cholesterol, weight, and body mass index. These outcome measures were determined by general practitioners, assisted by nurse practitioners during large-scale screening events at the university or at the workplace of the participants. The secondary outcomes were smoking, physical fitness, stages of behaviour change, medication use, and total cardiovascular risk. **Blood pressure** was measured with an Omron X6®. **Total cholesterol** was measured using capillary blood with an Accutrend Plus system®, **weight and body fat** was measured with a Tanita TBF-300A Body Composition Analyzer®. Body mass index (BMI) is body weight (kg) divided by the square of the height (m). **Smoking** was questioned using a short questionnaire from a national health questionnaire [18]. **Physical fitness** was tested with a step test, stepping up and down a platform (90 beats steps per minute) during 5 minutes [19]. Heart rate was recorded 1, 2 and 3 minutes after completing the step test in sitting position. Physical fitness level was calculated with the formula: 30.000 divided by 2 times the sum of the three recuperation hart rates. This continuous variable is transformed to a categorical variable that represents the fitness category as follows: < 55 (low); 55–64,9 (low average); 65–79,9 (high average); 80–89,9 (good); and > 90 (excellent). **Total cardiovascular risk** was determined using the score table, scoring participants with a risk ≥4% to die of an cardiovascular event as a high risk, 2-4% as medium risk and <2% as low risk [11]. The **stages of behaviour change** were measured using a single table for assessing physical activity, diet and quitting smoking [9]. For diet, for instance, a clear description of the dietary behaviour targeted was included in the questionnaire, namely: eating a low fat diet and 5 portions of fruit and vegetables daily.

### Statistical analyses

Repeated measures ANOVAs were used to examine time and time x study condition interaction effects. A p-level lower than 0.05 was considered to be significant. Evolutions of stages of change in the two study conditions were compared using Chi square analyses. All analyses were performed with SPSS 16.0.

## Results

### Participants

Overall 314 participants signed in to participate the study, 295 of these group underwent the multidisciplinary prevention consultation and were included (Figure 2). The mean age was 40.73 years (SD 10.54), 95 (32%) participants were female, 7 had a personal cardiovascular event in their personal history and 3 had diabetes (Table 1). There were no significant differences in baseline characteristics between MP and MLP except for smoking (21% in the MLP versus 11% in the MP arm, p < 0.05). Forty participants (13%) dropped out of the study prior to reaching the endpoint of the study. The main reason to stop was lack of time (n = 20); only interest in MP (n = 7), stopped as lawyer (n = 7) and disappointment in the study tools (n = 6). No one of the drop-outs had had a cardiovascular event. Contact was lost with 10 participants. There was no significant difference in drop-outs between the two study conditions. Participants who dropped out were significantly younger (p < 0.05), female (p < 0.05), without a family history of cardiovascular events (p < 0.05), having a significant lower BMI (p < 0.05) and lower daily unsaturated fat intake (p < 0.05). In total 255 participants (87%) completed the 3-year study.

**Table 1:** Baseline characteristics of the study population

**Baseline characteristics**	**Total (*n*** **= 295)**	**MP (*n*** **= 100)**	**MLP (*n*** **= 195)**	***P*-value difference between groups**
Gender (%)				
Male	200 (68)	67 (67)	133 (68)	0.83
Female	95 (32)	33 (33)	(62) (32)	
Mean age (y) (SD)	40.73 (10.54)	40.03 (10.57)	41.09 (10.54)	0.42
Mean systolic blood pressure (mmHg) (SD)	130.57 (19.87)	131.04 (18.96)	130.33 (20.36)	0.77
Mean diastolic blood pressure (mmHg) (SD)	83.76 (11.15)	84.12 (11.41)	83.58 (11.04)	0.69
Mean total cholesterol (mg/dl) (SD)	187.93 (30.66)	189.91 (32.60)	186.96 (29.65)	0.45
Mean body mass index (kg/m^2^) (SD)	24.95 (3.97)	24.92 (3.84)	24.97 (4.04)	0.93
Personal cardiovascular event (%)	7 (2)	4 (4)	3 (1.5)	0.19
Diabetes (%)	3 (1)	1 (1)	2 (1)	0.98
Smokers (%)	52 (18)	11 (11)	41 (21)	0.03
Mean physical fitness (SD)	55.34 (11.76)	54.97 (11.98)	55.54 (11.67)	0.72
Mean overall cardiovascular risk (SD)	0.01 (0.02)	0.01 (0.02)	0.01 (0.02)	0.89

*MP* Medical Program, *MLP* Medical + Lifestyle programme, *SD* standard deviation.

### Effectiveness of the cardiovascular prevention programmes

Among the study completers, a decrease in total cholesterol (p < 0.001), systolic blood pressure (p < 0.01), and diastolic blood pressure (p < 0.001) in both study conditions was found (Table 2). There was a significant increase in BMI (p < 0.01). Overall, there was a decrease in the fitness-score. Overall, the average fitness category was low average at the start of the study, and low at the end of the study. Most importantly, there was a significant decrease of total cardiovascular risk (p < 0.001). During the study period one participant in the MLP had a cardiovascular event. And one patient was diagnosed with diabetes in the MLP. There were no significant differences found between MP and MLP in blood pressure, cholesterolemia, BMI and Physical fitness (Table 2). At baseline, 48 participants (19%) were smoker compared to 29 (11%) at the study endpoint. The proportion of smoking cessation in the total sample was 44%. The proportion of smoking cessation in the MP (6/10 or 60%) versus the MLP (15/38 or 39.5%) was not statistically different.

**Table 2:** Repeated measures ANOVAs

	**Total sample (n = 255)**		**Mean change**	**F**_**time**_	**P values**	**MP (n = 82)**		**Mean change**	**MLP (n = 173)**		**Mean change**	**F**_time*condition_	**P values**
**Medical risk factor**	**Pre mean (SD)**	**Post mean (SD)**				**Pre mean (SD)**	**Post mean (SD)**		**Pre mean (SD)**	**Post mean (SD)**			
Systolic blood pressure (mmHg)	131.30 (18.30)	127.55 (16.52)	−3.75	10.71**	0.001	132.33 (19.41)	126.01 (18.75)	−6.32	130.82 (17.79)	128.28 (15.35)	−2.54	1.95	0.164
Diastolic blood pressure (mmHg)	84.39 (11.38)	79.77 (10.52)	−4.62	37.16***	0.000	85.37 (11.61)	79.15 (8.51)	−6.22	83.94 (11.27)	80.06 (11.34)	−3.88	1.99	0.159
Total cholesterol (mg/dl)	188.03 (30.79)	180.38 (30.92)	−7.65	15.33***	0.000	190.00 (33.00)	179.41 (27.61)	−10.59	187.09 (29.72)	180.84 (32.44)	−6.25	1.02	0.314
Body Mass Index (kg/m^2^)	25.14 (3.99)	25.53 (4.18)	0.39	11.96**	0.001	25.21 (3.85)	25.53 (3.84)	0.32	25.11 (4.07)	25.53 (4.34)	0.42	0.23	0.635
Physical fitness	55.21 (11.13)	53.79 (10.55)	−1.42	0.860	0.356	53.70 (8.45)	53.88 (8.95)	0.18	55.83 (12.07)	53.75 (11.20)	−2.08	1.22	0.273
Overall CVD risk^1^	0.011 (0.02)	0.008 (0.01)	−0.003	15.68***	0.000	0.011 (0.020)	0.008 (0.015)	−0.004	0.011 (0.015)	0.008 (0.012)	−0.002	0.95	0.332

^1^ Overall CVD risk is the 10-year risk of dying from a cardiovascular event using the SCORE risk tables.

* < .05; ** < .01; *** < .001.

### Medication use

A categorical variable was used for medication use with 4 possibilities: no use of medication, medication use decreased, medication use stayed the same, and medication use increased. Two hundred thirty seven patients used no medication. In 4 participants medication use decreased, in 16 participants the medication use stayed the same, and in 38 cases medication use increased. An interaction effect was found for systolic blood pressure (p < 0.001) and for diastolic blood pressure (p < 0.001) indicating that these decreases might be due to medication adherence. At the end of the study, in total, 33 patients took antihypertensive medication, 18 patients received anti-platelet medication, 29 used lipid lowering medication, and 4 patients used diabetes medication.

### Evolution of the stages of behaviour change

**Figure 3:**
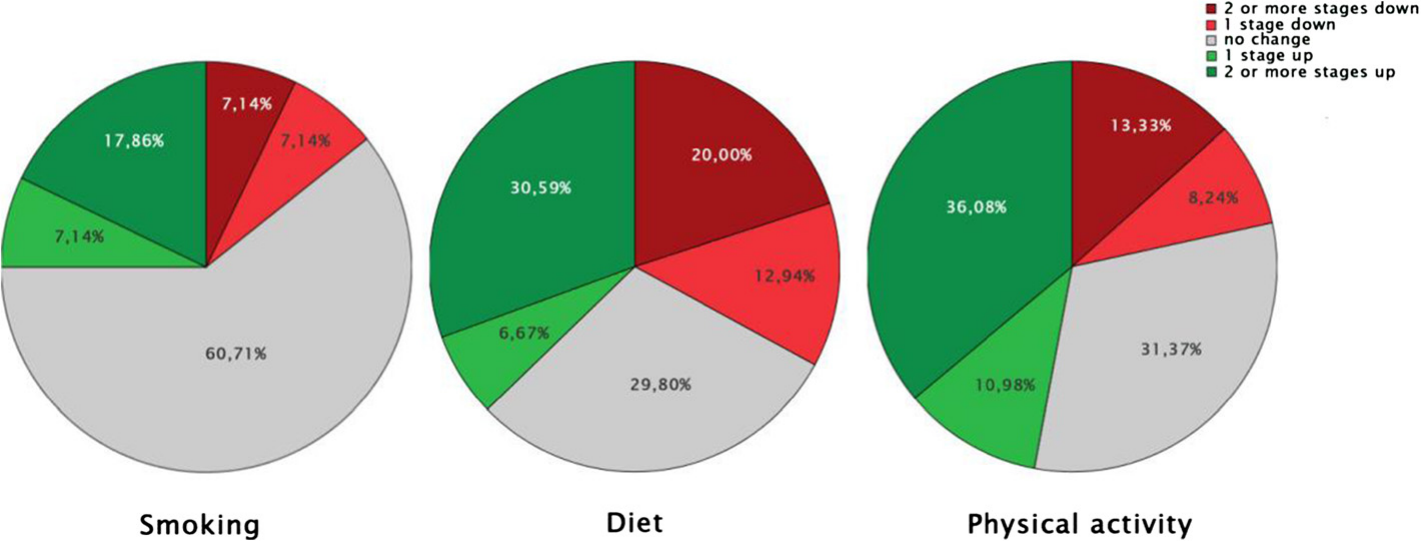
Stage of behaviour change for the 3 behaviours from baseline to follow-up.

No significant differences between the MP and MLP in evolution of the stages of behaviour change at the endpoint was found. In Figure 3 the evolution of the stage of behaviour change is given for the three behaviours: smoking cessation, diet and physical activity. For the total sample, the only difference from start to endpoint was found for the categories of change for smoking, not for physical activity or diet.

## Discussion and conclusion

In our population (relatively young and highly educated professionals) the combined medical and lifestyle programme (MLP) was not superior to the medical programme (MP). Both intervention programmes were effective in reducing medical cardiovascular risk factors and the overall CVD risk. This is an important finding given the achievements on a relatively short time period (3 years).

The MLP was not superior to the MP with regard to the medical risk factors and also with regard to the stages of behaviour change. These findings are in line with several recent reviews suggesting that multiple risk factor interventions including coaching methods produce only marginal results when implemented in general population samples [8]. Additionally, the lack of effect of the MLP compared to the MP might be due to several factors. Potential explanations for the lack of intervention effect are: the selection of relatively young and healthy highly educated professionals (70%), a medical assessment in both study conditions; the high level of choice options might have overwhelmed the participants and led them to select an insufficient intervention dose. Furthermore, it could be that the participants in the MP were involved in additional lifestyle programmes independent from PreCardio. So our results suggest that an intensive MLP is of modest value within a highly educated population sample, an organised MP within primary care can be sufficient to evoke improvements in risk factors. Medical screening and feedback on therapeutic goals within primary care can already lead to improvements with regard to risk factors such as cholesterol and blood pressure for a relatively young and highly educated sample.

The most important strength of this study was the use of a randomised design. The second strength is the inclusion of a mixed population sample in terms of overall CVD risk (high, medium and low CVD risk). However, the study has several limitations. A first limitation is the participants’ exposure to the lifestyle programme. Participants were free to choose the self-determined intervention intensity and the delivery mode of the MLP. This freedom was theory-inspired but led to some participants under using the programme which potentially limited the effect of the MLP. This might explain the lack of effect on the stages of behaviour change. The participants of the MLP received a mean intervention time of 22 minutes (SD 18) for diet and 37 minutes (SD 18) for physical activity [6]. Secondly, the participants were highly educated volunteers, limiting the generalisability of our findings. However, it is a general sample of active professionals and since population-targeted strategies are advised by the literature it is important to know which kind of interventions are cost-effective in this population subgroup. Nevertheless, secondary prevention initiatives are needed to be able to include effective and cost-effective interventions for people at high risk for CVD events such as acute coronary syndrome survivors [20]. Given their high motivation to take part in a cardiovascular prevention programme and their organisational and financial abilities to seek support elsewhere, it cannot be excluded that people allocated to the MP sought help to change their behaviour beyond the study context after receiving a printed profile with their risk factors.

### Effectiveness of prevention programs in general practice?

**STUDIES** which seek to improve the way preventive care is delivered in primary care are rather scarce and diverse (different study design, population and intervention(dose)) [8]. A study with a comparable population with regard to cardiovascular risk is ‘Hartslag Limburg’. It was a community based lifestyle programme to reduce cardiovascular risk factors in a cohort of 5 years [21]. All lifestyle factors improved. ‘Impala’, a nurse-led intervention study in primary care, didn’t benefit over the usual care mainly due to a study nurse detecting risk factors in both study conditions [22]. In ‘Euro action’ a nurse-coordinated, multidisciplinary, family based, ambulatory programme was studied [23]. In this programme healthier lifestyle scores and improved risk factors were obtained for patients with coronary heart disease and high risk patients in comparison to those followed by usual care. A comparable design study as the PreCardio-trial is the Swedish ‘Björknäs study’ [24]. Our findings are however not in line with their results: a significant difference comparing usual care and usual care plus a lifestyle programme. The superiority of their MLP could be explained by the high intensity of their lifestyle programme (weekly personal meetings by default, no self-selection) and patient selection (high risk). And most important the MP of our study was not comparable with usual care: it was a new multidisciplinary management model. This needs some explanation about the organization of **USUAL CARE** in Belgium. Until 2011 prevention was not remunerated in Belgium and prevention was performed exemplary. Usual care was organised around curative medicine meaning that a patient consulted a general practitioner only with physical complaints. GP performed a clinical examination with/without a blood sample. Based on these findings therapy was started. No medical or lifestyle prevention parameters (glycemie, cholesterolaemia, BMI, waist circumference, physical fitness, food intake, smoking status, …) were systematically detected nor treated. So primary prevention (to prevent the occurrence of disease and promote health) was scarce and secondary prevention (to prevent the progress of an illness or serious diagnosed risk factors) wasn’t organised. Since 1th of April 2011 Belgian government enlarged GP’s tasks with prevention (10 Euro per patient per year). But government forgot to define necessary criteria such as practitioner’s conditions & knowledge, goals, management, coordination of the prevention tasks. The under-evaluation of prevention is reflected in a disappointing follow-up of patient with CV risk factors in primary care (Euroaspire III survey conducted in 12 European countries) [25]. The results of the European surveys show that the lifestyle (of coronary and high-risk) patients is a major cause of concern, with persistent smoking and high prevalence of both obesity and central obesity. Blood pressure, lipids and glucose control are inadequate, with most patients, not achieving the targets defined in the prevention guidelines. There is considerable potential throughout Europe to raise the standard of preventive cardiology through more lifestyle intervention, control of other risk factors, and optimal use of prophylactic drug therapies. Cardiovascular disease prevention needs a comprehensive, multidisciplinary approach that addresses lifestyle and risk-factor management by cardiologists, general practitioners, nurses and other health professionals, and a healthcare system that invests in prevention.

### Implementation of prevention programmes in general practice on a large scale?

In most countries there is a need for a management model to organise prevention within usual care in collaboration with the governmental and academic actors. In such a model different aspects have to be discussed. The first aspect is the **SCREENING. Who will be screened?** A possibility could be to invite all adults between 40–75 years. It could be expected that in such a programme about half of the patients without a history of cardiovascular disease will receive at least one prescription, diet or health advice [26]. To perform the screening more cost-efficient, routine administrative data out of the electronic medical file could be used to invite people after a cardiovascular risk stratification [27]. In this case only the medium to high risk patient will be invited. **Who will perform the screening?** This could be a multidisciplinary screening consultation with a GP as prevention coordinator. The prevention coordinator sends the results to the treating GP for follow-up. In case of a multidisciplinary screening, the GPs will need (supporting) tools: administrative support, knowledge, sufficient time with an adequate remuneration, an electronic risk calculator, point of care testing for cholesterol-glycaemia, a weight balance and questionnaires on life style. These tools are necessary, it is already proven that GPs don’t always take time to give lifestyle advice or treat risk factors sufficiently. The next aspect is the **FOLLOW-UP. Who is going to invite the patient for follow-up:** high risk every 4 months, medium risk yearly and low risk every 3 years? The latter could be the task of the prevention coordinator in collaboration with the GP. **Who will perform the follow-up?** The follow-up will be performed by the treating GP (in collaboration with the cardiologist in case of secondary prevention). Also in the follow-up GPs will need the same (supporting) tools as mentioned above. This is in line with the quality improving factors of Buckley for management models in primary care (e.g., recall of patients and systematically monitoring of risk factors) [8].

This study showed that it is possible to implement a multidisciplinary management model in routine general practice with the incorporation of a medical prevention coordinator to perform the screening and to coordination. The follow-up can be done by the treating GP. This management model will improve patient follow-up regarding their risk factors.

## Competing interests

The authors declared that they have no competing interest.

## Authors’ contributions

NC: worked out the design of the study, coordinated the study, collected data, supervised statistics and drafted the manuscript. NJ: participated in the design of the study, carried out the study, collected data, supervised statistics and drafted the manuscript. EC: helped to draft the manuscript. WS: supervised statistics and gave intellectual feedback on the manuscript. ID: participated in the design of the study, supervised statistics and gave intellectual feedback on the manuscript. All authors read and approved the final manuscript.

## Pre-publication history

The pre-publication history for this paper can be accessed here:

http://www.biomedcentral.com/1471-2261/13/38/prepub
